# Role of a clinical pharmacist in managing diabetic nephropathy: an approach of pharmaceutical care plan

**DOI:** 10.1186/s40200-015-0213-7

**Published:** 2015-10-26

**Authors:** Amer Khan Mohammed, Chaitanya Medarametla, Mohammed Mahboob E. Rabbani, Kolamadhi Prashanthi

**Affiliations:** Department of Pharmaceutical Care, Raje Healthcare Pvt Ltd, My Health Pharmacy, 5-5-35/51B, plot no. 139, Hyderabad, 500072 Telangana India

## Abstract

**Aim:**

To evaluate the effect of low-protein diet on renal function in patient with diabetic nephropathy.

**Materials and methods:**

This is a case of a 57-year-old obese patient who is a known case of type 2 diabetes, hypertension, benign prostate hypertrophy and chronic kidney disease 4^th^ stage presented with the complaints of weakness, dyspnea, arthralgia, neuropathic pains and pedal edema which are prominent symptoms of Chronic kidney disease. Our healthcare team had visited patient’s home and analyzed the available reports on kidney profile, fasting sugar, post prandial sugar, HbA1c, lipid profile test and prescriptions which was found to be high. The glomerular filtration rate, serum creatinine and blood urea were 24 ml/min, 3.4 mg/dL and 90 mg/dL, fasting blood sugar, post prandial blood sugar and HbA1c were 226, 305 and 7.4 %, and total cholesterol and triglycerides were 145 & 95 respectively. Further discussion on diet, it came to know that the patient was on high carbohydrate diet. By considering the objective and subjective data, our team had done the assessment and come to a conclusion that high amount of carbohydrate diet with poor medication adherence had led to the hyperglycemia which developed diabetic nephropathy. We have recommended low protein, unsaturated fat, multivitamins, antioxidants and moderate carbohydrate diet. Two dietary assessment tools had been used in order to monitor patient’s adherence to the diet i.e. dietary record book and food frequency questionnaire.

**Results:**

We have carefully monitored the serum creatinine, glomerular filtration rate and blood urea for 12 months initially with an interval of 30 days for 3 months and later trimonthly up-to 12 months. Glomerular filtration rate was calculated by using the formula CKD-EPI creatinine equation. The values trend for first three months of serum creatinine and glomerular filtration rate were 2.8 mg/dL, 2.6 mg/dL,1.5 mg/dL and 24 ml/min, 26 ml/min, 51 ml/min respectively. Further, results has shown a significant improvement in the 6th, 9th and 12th month. The values of serum creatinine in the 6th, 9th and 12th month were 1.3 mg/dL, 1.1 mg/dL and 0.9 mg/dL, whereas golmerular filtration rate in the 6th, 9th and 12th month were 61 ml/min, 74 ml/min and 94 ml/min.

**Conclusion:**

The present study has demonstrated the protein diet restriction in-order to control the progression of renal failure. The dietary intervention on diabetic nephropathy plays a significant role in controlling the kidney failures. This is the first study, to our knowledge, to demonstrate the impact of pharmacist role in managing diabetic nephropathy by providing pharmaceutical care. Pharmaceutical care services should be encouraged in the community and hospital pharmacy which definitely plays a major impact in reaching the definite outcomes and providing higher quality of life.

## Background

The death and disability rate of chronic diseases have exceeded than communicable diseases comprising 49 %. In developing countries, the dominance of chronic diseases is not well recognized by healthcare professionals. The most common diseases are hypertension, angina, stroke, diabetes, thyroid diseases and chronic kidney disease [1].

According to international diabetes federation, India has 40.9 million diabetic patients in the year-2006, 50.8 million in 2010, 60 million in 2014 and it is expected to raise up-to 69.9 million by the year 2025. According to the epidemiological study in India, Hyderabad is known as diabetic capital of India with the prevalence of 16.6 % [2–5].

The dietary habits of Indians are 80 % carbohydrate and low omega-3-fatty acids which may cause insulin resistance leading to diabetic retinopathy, diabetic nephropathy, diabetic neuropathy and macro-vascular complications. Among them, diabetic nephropathy and diabetic retinopathy are most common [6, 7].

Though there is uncertainty about the etiology of kidney disease, heavy proteinuria is one the pathological aspect in diabetic nephropathy. A Study shows that restriction of protein helps to slow down the progression of albuminuria, glomerular filtration rate decline and occurrence of end stage renal disease [8].

Here, we discuss a patient with type 2 diabetes who developed diabetes nephropathy. We report that low protein diet in compliance with pharmaceutical care has improved the glomerular filtration rate.

In this case, we have evaluated the effect of low-protein diet on a patient with diabetic nephropathy which was found to be effective.

## Case presentation

This is a case of a 57-year-old obese patient who is a known case of type 2 diabetes, hypertension, benign prostate hypertrophy and chronic kidney disease 4^th^ stage presented with the complaints of weakness, dyspnea, arthralgia, neuropathic pains and pedal edema which are prominent symptoms of chronic kidney disease. Our healthcare team had visited patient’s home and analyzed the available reports on kidney profile, fasting sugar, post prandial sugar, HbA1c, lipid profile test and prescriptions which was found to be high. The glomerular filtration rate, serum creatinine and blood urea were 24 ml/min, 3.4 mg/dL and 90 mg/dL, fasting blood sugar, post prandial blood sugar and HbAIc were 226, 305 and 7.4 %, and total cholesterol and triglycerides were 145 & 95 respectively.

The patient was on metoprolol 50 mg once daily, amlodipine 5 mg once daily, telmisartan 40 mg once daily, torsemide 10 mg once daily and prazosin 5 mg once daily for managing blood pressure. His blood pressure was optimized i.e. 123/78 mmHg, ruling out hypertension as a cause for kidney damage. Ultra sonography abdomen has revealed grade – 1 prostatomegaly for which he was on tamsulosin 0.4 mg. Simultaneously he was also using tramadol and acetaminophen combination for arthralgia.

He was also using insulin 30 units, 15 units twice daily and voglibose 0.2 mg for diabetes with improper insulin storage. His lifestyle reveals that he consumes high amount of carbohydrate without any physical activity.

By considering the objective and subjective data, our team had done the assessment on the following parameters:

### Diet

The patient was on high carbohydrate diet which had led to dramatic increase in glycemic values causing diabetic nephropathy. Low protein, low potassium, low phosphorous, moderate carbohydrates, and high fiber diet have been recommended to the patient in order to control the blood glucose levels and progression of kidney failure. The dietary recommendations were based on the international recommendations of glycemic index and glycemic load [9].

We have also suggested a supplement which is rich in L-carnitine, choline, molybdenum, chromium, selenium, iodine, manganese, copper, zinc, iron, calcium, biotin, pantothenic acid, folic acid, niacinamide, vitamin B12, vitamin B6, vitamin B1, vitamin K, vitamin C, vitamin E, vitamin A, vitamin D, low protein, taurine, glycine, L-lysine, L-histidine and chloride which are essential nutrients and multivitamins for a kidney patient.

The kidney disease outcome quality initiatives (K/DOQI) nutrition guidelines suggest that a protein intake of 0.6 grams per kg of body weight may be beneficial when glomerular filtration rate (GFR) drops below 25, or approximately 25 percent remaining kidney function [10]. Where-as the American diabetic association suggests a, protein restriction of 0.8-1 g/kg/d may retard the progression of nephropathy [11–13].

Based on his weight (88 kg), we have recommended 52.8 grams protein per day. Our team had given a diet chart of low protein and moderate carbohydrate to the patient in order to control the abnormal values of blood glucose, serum creatinine and blood pressure.

### Dietary assessment

Two dietary assessment tools had been used in order to monitor patient’s adherence to the diet i.e. dietary record book and food frequency questionnaire.

- Dietary record book: day-wise dietary record book was given to the patient to monitor the dietary habits. Dietary record book was monitored bi-weekly by the pharmacist. Upon every visit, our pharmacist counselled and motivated the patient for better dietary adherence. Pharmacist also calculated the protein intake to make sure that it should not exceed than the recommended range.
- Food frequency questionnaire: Food frequency questionnaire (FFQ) has been evaluated every week in order to determine the highest and the lowest frequency of food items. Based on FFQ, dietary corrections had been made in accordance with glycemic index and glycemic load.

### Analgesics

Avoid oral analgesics as it may further damage the kidney function. We recommended him to use topical non-steroidal anti-inflammatory drugs instead of oral non-steroidal anti-inflammatory drugs.

### Storage of insulin

The patient was not aware of the fact that the insulin should be stored between 2 to 6 degrees which was getting saturated. We counselled him on proper storage of insulin.

### Brisk walking

We recommended him to do brisk walking regularly for 20 – 30 minutes every day.

## Result

Initially for 3 consecutive months with an interval of 30 days, we have monitored the laboratory values and diabetic diet. Later, patient was monitored trimonthly up-to 1 year. Glomerular filtration rate was calculated by using the formula CKD-EPI creatinine equation. The values trend for first three months of serum creatinine and glomerular filtration rate were 2.8 mg/dL, 2.6 mg/dL,1.5 mg/dL and 24 ml/min, 26 ml/min, 51 ml/min respectively. Further, results has shown a significant improvement in the 6th, 9th and 12th month. The values of serum creatinine in the 6th, 9th and 12th month were 1.3 mg/dL, 1.1 mg/dL and 0.9 mg/dL, whereas golmerular filtration rate in the 6th, 9th and 12th month were 61 ml/min, 74 ml/min and 94 ml/min. which can be shown in Table 1.

**Table 1:** Laboratory reports after pharmaceutical care

Parameter	1st month	2nd month	3rd month	6th month	9th month	12th month
Fasting blood sugar	108 mg/dl	90 mg/dl	73 mg/dl	84 mg/dl	92 mg/dl	89 mg/dl
Post prandial blood sugar	258 mg/dl	190 mg/dl	156 mg/dl	151 mg/dl	145 mg/dl	141 mg/dl
HbA1c	7.2 %	6.8 %	6.5 %	6.3 %	6.4 %	6.5 %
Serum creatinine	2.8 mg/dl	2.6 mg/dl	1.5 mg/dl	1.3 mg/dl	1.1 mg/dl	0.9 mg/dl
Blood urea	78 mg/dl	45.6 mg/dl	39 mg/dl	34 mg/dl	29 mg/dl	27 mg/dl
Glomerular filtration rate	24 ml/min	26 ml/min	51 ml/min	61 ml/min	74 ml/min	94 ml/min

The dietary habits was also been monitored by pharmacist personal visits. The diet was been assessed by using two dietary assessment tools such as dietary records and food frequency questionnaire. The objective of this assessment tools is to provide the diet with low protein and low to medium glycemic load and glycemic index.

Day-wise record book has been given to the patient in order to monitor the diet. Food frequency questionnaire (FFQ) has been evaluated every week in order to determine the highest and the lowest frequency of food items. Based on FFQ, dietary corrections had been made in accordance with glycemic index and glycemic load.

## Discussion

According to Hepler and Strand, pharmaceutical care is defined as “the responsible provision of medicine therapy for the purpose of a definite outcome that improves a patient’s quality of life” [14]. The concept of pharmaceutical care plan is to form the bridge between patient and physician by the pharmacist. Pharmaceutical care plan involves active co-ordination of the physician, patient and the pharmacist. The main challenge for the pharmacist is to record the subjective and objective data, to understand the chronic condition of the patient, to counsel him on the lifestyle and diet and if necessary, recommendation to physicians with an appropriate evidence based literature.

In this case, due to in-science the patient was following high carbohydrate and low omega-3 fatty acid diet. This composition is present in all the meals throughout the day. Both high carbohydrate and low omega-3 fatty acid lead to diabetic complications diet. Later, patient developed the microvascular complication of diabetes that is diabetic nephropathy.

According to a systemic review conducted by Salgado TM, pharmacist played an important role in achieving the quality outcomes in patients with chronic kidney disease by reducing all-cause hospitalizations, incidence of end stage renal disease or deaths with diabetic nephropathy and managing anemia, blood pressure, lipid profile etc. In our patient, we have not only reduced the incidence of hospitalization, end stage renal disease but also managed other health care parameters like diabetes, blood pressure and cholesterol [15].

Unfortunately Indian community pharmacies are only involved in dispensing the medicines. Complete knowledge of drug-drug interactions, dose based on patient biochemical data and fed-fast variability of drug bioavailability would help both patient and physician. But, community pharmacy has a great opportunity to improve the health-care among the community population. However, the pharmacist’s role in India is expected to standardize the pharmacy settings which not only sells the medicines but also provides the services.

Nowadays, pharmaceutical care has become a dominant form of practice for thousands of pharmacists all over the world. However, for the patients and pharmacists in India, this concept remains unknown because it is not being performed as a routine, and pharmacists especially community pharmacists are not aware of the same. It is important to emphasize the fact that pharmaceutical care is aimed to achieve rational and evidence-based pharmacotherapy, which is beneficial for patient and society.

This case study is a testimony of critical role of pharmacist in patient care. Our team suggested relevant diet to the patient that improved the clinical status of patient by reducing the biomarkers such as serum creatinine and blood urea.

According to tumkur et.al, Indian pharmacists’ are underutilized in the management of patient’s health. In India, patients’ therapy is only managed by physician with the help of nurses which doesn’t give an opportunity to the pharmacist. But, pharmacist has a very vast role in managing patients’ health. Retail pharmacy is best suited to provide the individualized patient care by providing patient counseling, reviewing of prescriptions, detection of potential side effects, etc.

Unfortunately, Indian pharmacy education is more industrial than clinical, which requires the Indian amendment to include the concept of pharmaceutical care in various communities [16].

## Conclusion

The present study has demonstrated the protein diet restriction in-order to control the progression of renal failure. The dietary intervention on diabetic nephropathy plays a significant role in controlling the kidney failures. This is the first study, to our knowledge, to demonstrate the impact of pharmacist role in managing diabetic nephropathy by providing pharmaceutical care. Pharmaceutical care services should be encouraged in the community and hospital pharmacy which definitely plays a major impact in reaching the definite outcomes and providing higher quality of life. There is a vast scope for pharmaceutical care and future studies should be carried out to determine its impact. Knowledge of pharmaceutical care should be reached out to patients’ as well as physicians, in order to bridge the gap between them. However, it would be necessary to continue evaluating kidney function in more subjects in order to confirm whether the positive effect of the present intervention can be sustained.

My health pharmacy is the first organized retail chain in India which has introduced the concept of pharmaceutical care in managing the patients’ health.

## Consent

Written informed consent was obtained from the patient for publication of this case report.
